# Standing transthoracic echocardiography: a feasibility study

**DOI:** 10.1186/s44156-025-00075-2

**Published:** 2025-05-20

**Authors:** Stephen P Juraschek, Noelle Ojo, Janet Monroe, Jordan B Strom, Jessica Stout, Warren J Manning, Ruth-Alma N. Turkson-Ocran, Gabrielle Kolaci, Kaitlynn Geier, Carla Baptista, Araina Picanzo, Kenneth J Mukamal, Jason D Matos

**Affiliations:** 1 Division of General Medicine, Section for Research, Beth Israel Deaconess Medical Center, 330 Brookline Avenue, CO-1309, #217, Boston, MA 02215 USA; 2 Department of Medicine, Brigham and Women’s Hospital, Harvard Medical School, Boston, MA USA; 3 Richard A. and Susan F. Smith Center for Outcomes Research in Cardiology, Beth Israel Deaconess Medical Center, Boston, MA USA; 4 Division of Cardiology, Beth Israel Deaconess Medical Center, Boston, MA USA; 5 Department of Radiology, Beth Israel Deaconess Medical Center, Harvard Medical School, Boston, MA USA

**Keywords:** Autonomic dysfunction, Supine, Standing, Blood pressure, Transthoracic echocardiogram, Orthostatic hypotension

## Abstract

**Background:**

Orthostatic hypotension (OH) is associated with cardiovascular disease, particularly among older adults. While a standing transthoracic echocardiogram (TTE) could theoretically identify changes in cardiac output to diagnose cardiogenic OH, there are no established protocols for orthostatic TTEs and their feasibility is unknown.

**Methods and results:**

We recruited 115 patients scheduled for elective outpatient TTE. Consenting participants, who were able to stand safely, underwent their scheduled recumbent TTE, followed by a standing TTE, performed within 1–2 minutes of standing. The focused TTE used the apical window to measure velocity time integral across the aortic valve to assess cardiac output. Blood pressure (BP) was measured in the supine and standing positions and patients were asked about symptoms of dizziness and lightheadedness. OH was defined as a change in standing minus supine systolic BP ≤-20 mm Hg or in diastolic BP of ≤-10 mm Hg. Of the 115 enrolled participants, 102 (89%) completed the standing echocardiogram protocol. Among those completing, mean age was 63.4 (SD, 14.8) years (38% were ≥ 70 years), 48% women, and 34% had a BMI ≥ 30 kg/m^2^. There were 21% with OH. Upon standing, systolic BP changed by -5.9 mm Hg (95% CI: -9.5, -2.2), diastolic BP by 2.4 mm Hg (-0.1, 4.8), and cardiac output by -0.4 L/min (95% CI: -0.7, -0.1). Change in cardiac output (per 1 L/min) was associated with a higher odds of systolic OH (OR: 1.60; 95% CI: 1.05, 2.42), but not diastolic OH (OR: 1.21; 95% CI: 0.63, 2.32).

**Conclusions:**

Standing TTE is safe, well-tolerated, and feasible in the ambulatory setting. Moreover, TTE changes in cardiac output are associated with systolic OH. This clinical assessment shows promise for distinguishing OH etiologies and could inform further research on treatments to prevent OH.

**Supplementary Information:**

The online version contains supplementary material available at 10.1186/s44156-025-00075-2.

## Introduction

Orthostatic hypotension (OH) is common among adults with hypertension and heart failure, and is associated with a higher risk of cardiovascular disease events [1]. Although the autonomic reflex in response to standing is neurally-mediated, both beta and alpha adrenergic responses play critical roles in maintaining blood pressure (BP) in the upright position [2, 3]. While OH has traditionally been viewed through a lens of neurodegenerative disease, there is increasing recognition that cardiovascular conditions affecting cardiac output (i.e., impaired heart rate augmentation) may also contribute to OH [4, 5]. Moreover, there is growing evidence that orthostatic hypertension, a rise in blood pressure after standing, is strongly associated with cardiovascular disease events, [6, 7]. but little is understood about how heart function after standing might contribute to this pathologic hypertensive phenotype. Furthermore, current approaches to diagnose OH, active stand or tilt table testing, do not assess orthostatic changes in heart function after standing.

While transthoracic echocardiography (TTE) is the primary modality for characterizing cardiac structure and function, it is traditionally performed in the supine or recumbent position for both resting and post-exercise assessments [8, 9, 10]. This position is preferred to permit unobstructed view of the left ventricle (LV) across a range of body types, while permitting for repositioning in cases of challenging viewing windows. While there is precedent for other forms of standing radiographic assessments (e.g., x-ray), [11] and historic stress TTE, [12, 13] there have been few reports to describe the performance of echocardiogram in the standing position and how parameters might relate to orthostatic changes in BP [14, 15, 16]. TTE in the standing position could allow for an assessment of a major mechanism of the autonomic response to standing, i.e., cardiac augmentation, which could potentially inform if OH had a cardiogenic origin.

Our objectives in this pilot study were to (1) characterize the performance, quality, and safety of a standing TTE protocol among a general outpatient population as well as older and obese adults, (2) examine TTE parameters potentially related to change in BP, and (3) determine whether changes in cardiac output determined using a standing TTE protocol were associated with OH or orthostatic hypertension (i.e., a drop or rise in systolic BP of at least 20 mm Hg or diastolic of at least 10 mm Hg, respectively). We hypothesized that a standing TTE would be safe, efficient, and informative and that blunted cardiac output upon standing would be associated with OH while elevated cardiac output upon standing would be associated with orthostatic hypertension.

## Methods

### Study design and population

In this prospective feasibility study, we enrolled a convenience sample of patients referred for elective outpatient TTE at Beth Israel Deaconess Medical Center, an academic practice, over two periods (1/31/22 to 2/18/22 and 12/5/22 to 12/9/22) based on the availability of echocardiographers. Patients were contacted during or the week prior to their appointment and recruited consecutively without accounting for their TTE indication. We excluded patients with a history of irregular heart rhythm or prosthetic valves. Written informed consent was obtained in the echocardiography suite, immediately prior to testing. The Institution Review Board at Beth Israel Deaconess Medical Center provided ethical approval for this study protocol.

## Supine and standing transthoracic echocardiogram

Cardiac sonographers performed a scheduled supine TTE, using Vivid e95 of S70 (General Electric Healthcare, Chicago, Illinois) and X5 transducer for 2-dimensional and Doppler acquisitions. Following guidelines from the American Society of Echocardiography (ASE), 2-dimensional methods were used to obtain standard images to determine aortic valve pulse wave velocity time integral (LV outflow track [LVOT]), LVOT diameter, and heart rate (based on the aortic valve at the time of the image acquisition). Once the clinical TTE was complete, the patient was asked to stand up and position their arm on a bedside table, pre-positioned to support their outstretched arm at a 70–80 degree angle from their torso. The protocol attempted to capture acute changes in heart function by performing the standing TTE within 2 min of standing.

Once the participant was in the standing position, the sonographer recorded a blank image to signal the start of the standing TTE. Duration of the standing TTE was determined based on the time required to capture desired images minus the time stamp of the blank image. The sonographer would work to optimize their viewing window with their probe to document LVOT pulse wave velocity time integral. We estimated cardiac output using the LVOT diameter and the velocity time integral, using the equation: Cardiac Output = π*[LVOT diameter/2]^2^ * [pulse wave velocity time integral]* [heart rate] [17].

The sonographer was asked to document reasons the standing TEE was not performed or suboptimal (if performed), specifically due to poor supine image, poor standing image, lost viewing window (i.e., no image), patient safety, or any other reason. The cardiologist graded Doppler quality using a 5-point scale, 1 being the worst possible and 5 being the best (clearest) possible.

All TTEs were interpreted by a Level III-trained cardiologist certified by the National Board of Echocardiography. Intra-observer agreement for the parameters above were evaluated by randomly selecting 10% of the sample (*N* = 11 images) for re-review in a blinded manner by the primary cardiologist. Factors for selection included sex, age over 70 years, obesity, and hypertension status. The Spearman’s correlation coefficient was 0.50 for LVOT diameter, 0.94 for supine pulse wave velocity time integral, 0.94 for supine heart rate, 0.97 for standing pulse wave velocity time integral, and 0.99 for standing heart rate. Inter-observer agreement for the parameters above was evaluated by randomly selecting 10% of the sample (*N* = 11 images) for independent review by a second cardiologist, using the same factors for selection above. The inter-observer agreement based on Spearman’s correlation coefficient was 0.40 for LVOT diameter, 0.89 for supine pulse wave velocity time integral, 0.99 for supine heart rate, 0.81 for standing pulse wave velocity time integral, and 0.97 for standing heart rate.

## Supine and standing blood pressure

Prior to their TTE, the sonographer would adjust a bedside table to each patient’s height to ensure their arm could rest at a 70–80 degree angle from their torso. They were then positioned in the supine position and underwent a single blood pressure measurement. The cuff was placed over bare skin over the right arm and measured with an Omron Hem907XL (Omron Electronics, Hoffman Estates, IL) [18] to record a single systolic blood pressure, diastolic blood pressure, and heart rate. A rest period was not required prior to measurement.

After the supine TTE was complete, participants were asked to stand and place their arm on the adjacent prepositioned table (note sometimes sonographers used other clinic furniture to achieve the appropriate angle). As soon as their arm was rested and they were standing a single standing blood pressure assessment was performed. After the standing focused TTE was complete, patients were subsequently asked: “Did you experience any discomfort during the standing procedure (4-point scale, none 0, minimal 1, moderate 2, severe 3)?” and “Did you feel dizziness, lightheadedness, faint, or like you might black out in the process of standing up (4-point scale, none 0, minimal 1, moderate 2, severe 3)?” [19].

Orthostatic hypotension was defined as a drop in systolic blood pressure of ≥ 20 mm Hg or diastolic blood pressure of ≥ 10 mm Hg when moving from supine to standing, while orthostatic hypertension was defined as a rise in systolic blood pressure of ≥ 20 mm Hg or diastolic blood pressure of ≥ 10 mm Hg when moving from supine to standing. We also examined the following definitions: systolic or diastolic components of OH or orthostatic hypertension, based on either the rise or drop in systolic or diastolic blood pressure, respectively.

## Other covariates

We extracted the following information from the medical record: age, sex, race, ethnicity, TTE indication (valvular disease, heart failure, coronary disease, arrhythmia, or other), hypertension status, diabetes (history or glucose lowering medication), or history of cardiovascular disease (coronary artery disease, heart failure, or stroke/transient ischemic attack). We also extracted most recent weight and height prior to the echocardiogram to derive body mass index. In addition, we documented any blood pressure medications.

### Statistical analysis

Characteristics of the consented population and those who completed the standing protocol were summarized via means and proportions. We also summarized protocol performance metrics overall and among older participants (age ≥ 70 years) and among participants with a body mass index ≥ 30 kg/m^2^) via means and proportions.

We determined mean blood pressure, pule wave velocity time integral, heart rate, and cardiac output in both supine and standing positions and compared change (standing minus supine) values via t-tests. We also used Spearman’s correlation coefficient to compare differences in the TTE measures and with LVOT diameter (only assessed in the supine position). Lowess curves and scatter plots were used to visualize the relationship between cardiac output and change in systolic or diastolic blood pressure.

We examined the association between change in cardiac output (standing minus supine) with OH, systolic OH, diastolic OH as well as orthostatic hypertension, systolic orthostatic hypertension, and diastolic orthostatic hypertension using logistic regression with adjustment for age and sex. We also examined the relationship between change in cardiac output with change in systolic or diastolic blood pressure as well as supine or standing values via linear regression models adjusted for age and sex.

In secondary analyses, we looked at components contributing to cardiac output estimates, namely, LVOT, change in velocity time integral, and change in heart rate with respect to OH and changes in blood pressure. These analyses were similarly performed using logistic regression for binary dependent variables or linear regression for continuous dependent variables adjusted for age and sex. In addition, we performed a sensitivity analysis examining the relationship between orthostatic changes in blood pressure with change in cardiac output as the dependent variable. Note, in a few cases, sex predicted the diastolic OH outcome perfectly, reducing the sample contributing to the model. In these cases, a model without adjustment for sex was performed. Finally, we examined common antihypertensive medication use in relation to changes in blood pressure or TTE measures using linear regression adjusted for age and sex.

All analyses were conducted with Stata 15.1 (StataCorp LP, College Station, TX). A two-tailed *P*-value < 0.05 was considered statistically significant.

## Results

### Population characteristics

**Fig. 1:**
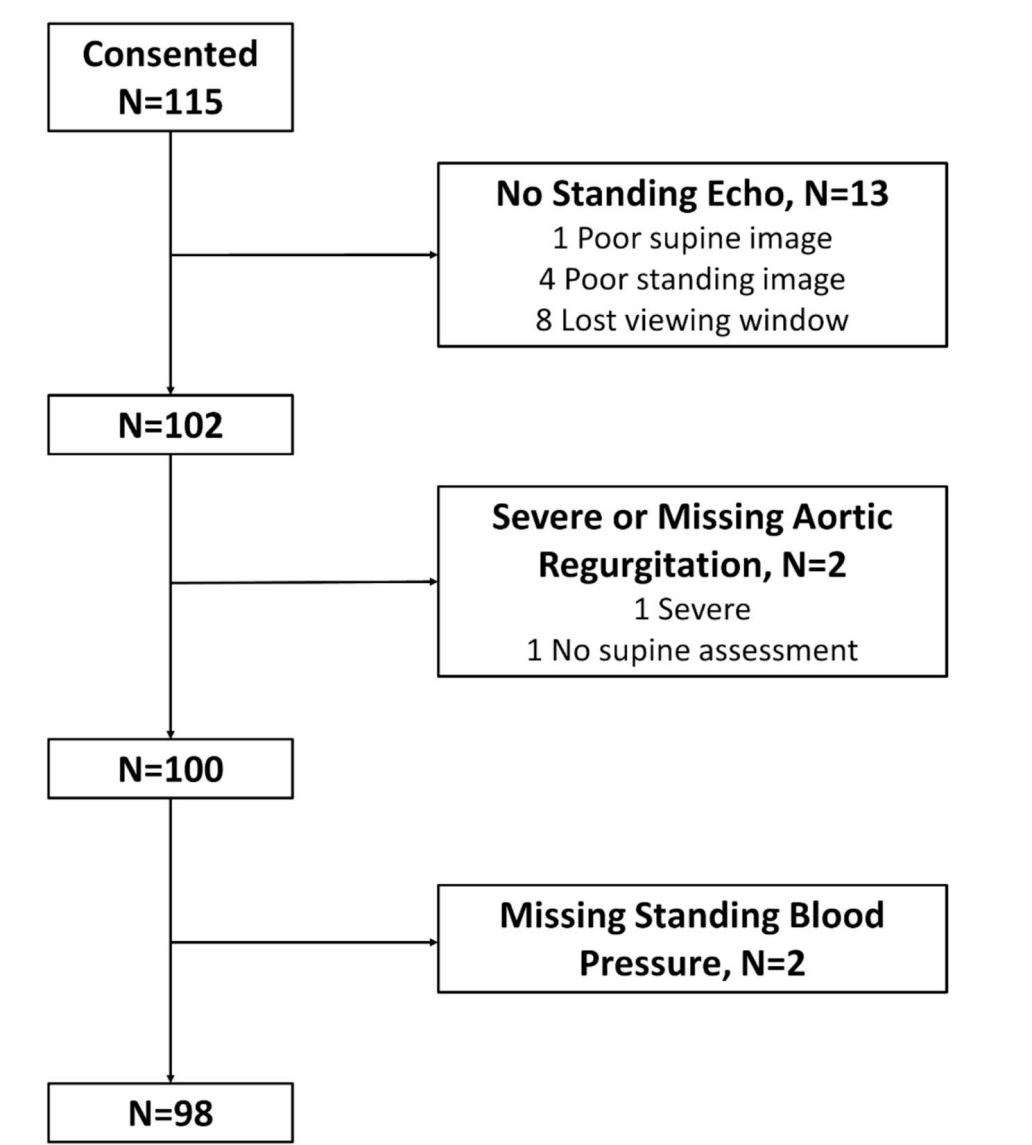
Flow diagram of participants that consented and ultimately contributed to the analytic sample. All patients undergoing elective transthoracic echocardiogram were invited to participate except patients with a history of irregular heart rhythm or prosthetic valves

Of the 115 consenting patients (mean age 63.7 years [SD, 14.6], 50% female and 75% self-identifying as White), 63% of those referred had a history of hypertension and 36% had a history of cardiovascular disease (Table 1). Indications for TTE varied, but arrhythmia and valvular disease represented 22% and 16% of referrals, respectively. Of those referred, 98 (85%) contributed data to our analytic sample (Fig. 1). Characteristics of the 98 included in our analytic sample were similar to characteristics of the referred population.

**Table 1:** Baseline characteristics

		Referred population		Population completing protocol	
		*N*	Mean (SD) or %	*N*	Mean (SD) or %
Age, yr		115	63.7 (14.6)	98	63.4 (14.8)
Age 70 year and older, %		115	37	98	38
Female, %		115	50	98	48
White race, %		115	75	98	79
Not Hispanic or Latino, %		115	70	98	74
Body mass index, kg/m^2^		105	29.0 (5.8)	89	28.5 (5.3)
Body mass index ≥ 30 kg/m^2^, %		105	37	89	34
Hypertension, %		114	63	97	62
Diabetes, %		114	11	97	12
Cardiovascular disease, %		113	36	96	38
Echo indication, %					
	Valvular Disease	114	16	97	17
	Heart Failure	114	5	97	5
	Coronary Disease	114	6	97	6
	Arrhythmia	114	22	97	25
	Other	114	51	97	47
ACE/ARB, %		115	44	98	46
Beta blocker, %		115	37	98	41
Thiazide or loop diuretic, %		115	23	98	18
Calcium channel blocker, %		115	23	98	24
Mineralocorticoid receptor antagonist, %		115	3	98	2
Nitrate*, %		115	2	98	2
Vasodilator, %		115	1	98	1
Orthostatic hypotension, %		102	22	98	21
Systolic orthostatic hypotension, %		102	19	98	18
Diastolic orthostatic hypotension, %		102	10	98	10
Orthostatic hypertension, %		102	25	98	22
Systolic orthostatic hypertension, %		102	7	98	5
Diastolic orthostatic hypertension, %		102	24	98	21

Abbreviations: ACE/ARB, angiotensin converting enzyme inhibitor or angiotensin II receptor blocker; SD, standard deviation. Note there were no patients actively prescribed an alpha-1-blocker at the time of the echocardiogram

*Isosorbide mononitrate

## Process and safety

Of the 115 referred, 90% successfully had a focused TTE performed and 89% had a standing blood pressure (Table 2). On average, the protocol took 94.1 s (SD, 75.1). Among the 103 with standing images, Doppler quality was moderate to best (i.e., 3 to 5 with 5 being most clear) in 87% of cases. Of the 15 with difficulties performing the protocol, standing images were not obtained for 13 patients. The most common reason TTE was not completed include lost viewing window (9 of 15 or 60%) followed by poor standing image (4 of 15 or 27%). Among the 82 who answered questions related to discomfort and dizziness, only 5% reported any discomfort with the protocol and only 7% expressed any dizziness in the process of standing.

**Table 2:** Process and safety, all participants

		Mean (SD) or %					
		*N*	Overall, % or Mean (SD)	*N*	Age ≥ 70 years, % or Mean (SD)	*N*	Body Mass Index ≥ 30 kg/m^2^, % or Mean (SD)
Supine blood pressure performed, %		115	90	43	86	39	85
Standing blood pressure performed, %		115	95	43	88	39	95
Standing focused TTE performed, %		115	89	43	86	39	82
Duration, seconds		96	94.1 (75.1)	36	107.8 (80.2)	30	102.4 (79.9)
Severity of aortic regurgitation							
	0+	102	84	37	78	33	91
	1+	102	14	37	22	33	9
	1–2+	102	1	37	0	33	0
	2+	102	1	37	0	33	0
Doppler quality (scale 1–5, 5 best)							
	1 (Worst)	103	3	37	14	33	6
	2 (Poor)	103	10	37	32	33	6
	3 (Moderate)	103	36	37	30	33	33
	4 (Good)	103	33	37	24	33	36
	5 (Best)	103	18	37	0	33	18
Reason TTE not completed, %*							
	Poor supine image	15	13	6	33	8	25
	Poor standing image	15	27	6	67	8	13
	Lost or no viewing window	15	60	6	0	8	63
	Patient safety	15	0	6	0	8	0
Discomfort, %**		82	5	32	6	28	0
Dizziness, %**		82	7	32	0	28	0

Abbreviations: TTE, transthoracic echocardiography

*2 of these had a TTE performed despite difficulties: 1 with a poor supine image and 1 with a lost viewing window

**Discomfort and dizziness questions were only collected for 82 participants

Process and safety was evaluated in two pre-specified groups: adults age 70 years and older and adults with obesity (body mass index ≥ 30 kg/m^2^). While completion rates were generally comparable among older adults, the protocol took slightly more time (mean 107.8 s, SD, 80.2). Moreover, Doppler quality was moderate to good in 54%. Four did not have the standing echocardiogram completed due to poor standing quality and of the 32 asked about discomfort or dizziness, 6% expressed discomfort with the procedure, while 0% experienced dizziness upon standing.

Completion rates were also high among participants with obesity at 85%. Image quality was moderate to best among 87% of participants with obesity. Difficulty completing the protocol was reported among 8 patients with obesity, with 5 being due to a lost viewing window and 1 due to a poor standing image.

### Body position and change in blood pressure and echocardiogram measures

Of the 98 participants who completed the entire standing protocol (both TTE and blood pressure), systolic blood pressure went down 5.9 mm Hg (95% CI: -9.5, -2.2; *P* = 0.002), while diastolic blood pressure did not change (difference was 2.4 mm Hg; 95% CI: -0.1, 4.8; *P* = 0.059) (Table 3). On average, pulse wave velocity time integral was lower after standing (-4.0 units; 95% CI: -5.0, -3.0; *P* < 0.001) and heart rate was increased (6.8 beats per minute; 95% CI: 5.0, 8.7; *P* < 0.001). Cardiac output went down with standing by 0.4 L/min (95% CI: -0.7, -0.1; *P* = 0.004).

**Table 3:** Physiologic measurements by position, *N* = 98

	Supine	Standing	Difference (Standing - Supine)	
	Mean (SD)	Mean (SD)	Mean (95% CI)	P*
Systolic blood pressure, mm Hg	130.7 (20.5)	124.8 (23.4)	-5.9 (-9.5, -2.2)	0.002
Diastolic blood pressure, mm Hg	70.7 (10.9)	73.0 (12.7)	2.4 (-0.1, 4.8)	0.059
Left ventricular outflow tract diameter, cm	2.1 (0.2)	-	-	
Pulse wave velocity time integral	23.2 (6.1)	19.2 (5.4)	-4.0 (-5.0, -3.0)	< 0.001
Heart rate, beats per minute	68.8 (12.2)	75.7 (14.8)	6.8 (5.0, 8.7)	< 0.001
Cardiac output, L/min	5.1 (1.4)	4.8 (1.7)	-0.4 (-0.7, -0.1)	0.004

Abbreviations: CI, confidence interval; SD, standard deviation

*Statistical comparison via t-tests

### Cardiac output and orthostatic changes in blood pressure

**Fig. 2:**
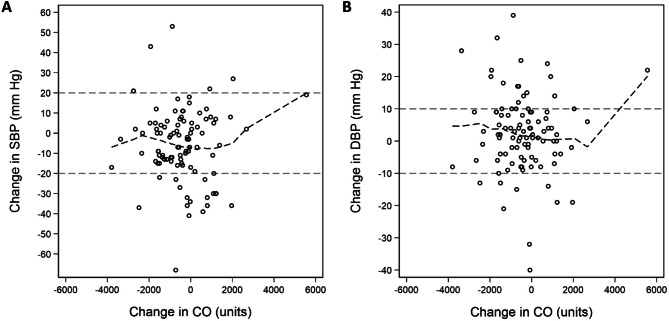
Scatter plots of change in cardiac output (CO) and change in systolic blood pressure (SBP) **(A)** and diastolic blood pressure (DBP) **(B)**. The dashed line represents a Lowess curve. The gray horizontal dashed lines represent threshold used to define systolic or diastolic orthostatic hypotension or hypertension (systolic ± 20 mm Hg or diastolic ± 10 mm Hg, respectively)

Higher change in cardiac output (per 1 L/min) was not significantly associated with a higher odds of orthostatic hypertension (OR 1.45; 95% CI: 0.98, 2.13; *P* = 0.062), but was associated with systolic OH (OR 1.60; 95% CI: 1.05, 2.42; *P* = 0.027) (Table 4). A higher cardiac output was not associated with diastolic OH, orthostatic hypertension, or orthostatic changes in blood pressure. In general, higher cardiac output was associated with lower supine and standing blood pressure, although not significantly. When examined via a Lowess curve, an increase in cardiac output upon standing seemed associated with a drop in BP, but these patterns were not strong (Fig. 2).

**Table 4:** Association of change in cardiac output (per 1 L/min) with orthostatic changes in blood pressure, *N* = 98*

Dependent variable	β (95% CI) or OR (95% CI)	*P*
Orthostatic hypotension	1.45 (0.98, 2.13)	0.062
Systolic orthostatic hypotension	1.60 (1.05, 2.42)	0.027
Diastolic orthostatic hypotension*	1.21 (0.63, 2.32)	0.57
Orthostatic hypertension	0.98 (0.68, 1.41)	0.90
Systolic orthostatic hypertension	0.90 (0.47, 1.72)	0.74
Diastolic orthostatic hypertension	1.07 (0.74, 1.54)	0.73
Change in systolic blood pressure (supine minus standing), mm Hg	-0.29 (-2.97, 2.39)	0.83
Change in diastolic blood pressure (supine minus standing), mm Hg	-0.33 (-2.20, 1.55)	0.73
Supine systolic blood pressure, mm Hg	-1.87 (-4.74, 1.01)	0.20
Supine diastolic blood pressure, mm Hg	-1.22 (-2.89, 0.46)	0.15
Standing systolic blood pressure, mm Hg	-2.15 (-5.66, 1.35)	0.23
Standing diastolic blood pressure, mm Hg	-1.54 (-3.45, 0.36)	0.11

Abbreviations: CI, confidence interval; OR, odds ratio

Adjusted for age, female

*Sex predicts dependent variable perfectly, which resulted in a reduced *N* = 47 for regression. In a model without adjustment for sex, the diastolic orthostatic hypotension OR (95% CI) was 1.02 (0.63, 1.65) for *N* = 98

### TTE measures and orthostatic changes in blood pressure

Changes in cardiac output and its contributing variables (i.e., heart rate, pulse wave velocity time integral, and LVOT diameter) were not strongly correlated with orthostatic changes in blood pressure (Spearman’s coefficients between − 0.18 and 0.17) (Supplement Table ST1). Only change in heart rate was associated with systolic OH (OR 1.08; 95% CI: 1.02, 1.16; *P* = 0.016) (Supplement Table ST2-ST3). Otherwise, there was no clear relationship between changes in heart rate, pulse wave velocity time integral, and LVOT diameter with OH or changes in blood pressure.

### Cardiac output as outcome

In sensitivity analyses, we examined orthostatic changes in blood pressure with cardiac output. Similar to the models above, only systolic OH (versus no systolic OH) was associated with a higher cardiac output of 0.77 (95% CI: 0.08, 1.46; *P* = 0.030) (Supplement Table ST4).

### Antihypertensive medications

In general, antihypertensive medications were not associated with changes in blood pressure or TTE measures (Supplement Table ST5); although, using an angiotensin converting enzyme inhibitor or angiotensin II receptor blocker was associated with a higher change in diastolic blood pressure (5.32 mm Hg; 95% CI: 0.22, 10.43; *P* = 0.041) and using a thiazide or loop diuretic was associated with a higher change in velocity time integral (2.87; 95% CI: 0.20, 5.53; *P* = 0.035).

## Discussion

In this population of patients referred for elective ambulatory TTE, a standing focused TTE protocol was successfully performed in the majority of participants independent of age or obesity status with most images revealing moderate or better Doppler quality. The procedure was quick, well-tolerated, and safe. There were significant differences in systolic blood pressure, pulse wave velocity time integral, heart rate, and cardiac output with standing. Moreover, an increase in cardiac output after standing, driven by heart rate (not stroke volume), was associated with a higher odds of systolic OH. These results suggest that a standing focused TTE is feasible and potentially useful for distinguishing mechanisms responsible for the cardiac response and its contribution to OH.

In the normal state, BP is maintained after standing due to the immediate action of the autonomic nervous system. Baroreceptors in the carotid arteries and the aorta detect the drop in pressure after standing, triggering vasoconstriction (α-adrenergic response) and increased heart rate (β-adrenergic response) [20]. Moreover, compression of splanchnic veins and contraction of skeletal muscles increase venous return, augmenting cardiac output [2, 3]. Impairment in these response pathways contribute to OH, which is strongly associated with a number of adverse events in older adults, including falls, cardiovascular disease, stroke, dementia, and death [1, 21, 22, 23]. These associations are thought to be mediated by prolonged hypoperfusion of muscle, heart, and brain due to a deficient autonomic response to gravity. However, we recently demonstrated that OH is also strongly related to subclinical cardiac damage, strain, and future risk of cardiovascular events [1]. Furthermore, in prior work we showed that antihypertensive agents that affect heart rate (i.e., beta blockers) increase risk of OH [5]. However, the relationship between orthostatic changes in cardiac output with orthostasis are poorly characterized. A number of cardiac conditions, i.e., valvular disease, arrhythmia, and heart failure, have been related to various components of OH and its sequelae [5, 24, 25, 26, 27, 28]. Conventional supine TTE with Doppler flow across the aortic valve readily provides information on stroke volume and cardiac output. Acquisition of this information in the standing position would allow for assessment of changes that could identify occult cardiac dysfunction uncovered by the standing position.

Two populations of concern for standing focused TTE are older adults and adults with obesity, related to patient safety, viewing windows, and quality of the standing image [29, 30]. Our protocol revealed minimal safety concerns. While there was some discomfort and dizziness reported, these were rare occurrences. Among older adults, there were no issues with maintaining a viewing window, although at times the Doppler quality was poor. Among obese adults, while a lost viewing window was more common, it was also generally rare and the quality of the standing image and Doppler tended to be quite good. These findings suggest that this protocol may be both safe and successful among these important patient groups.

Despite our small pilot sample, change in cardiac output was associated with systolic OH. Furthermore, our study illustrates how TTE might be used to elucidate the cause of the change in cardiac output, particularly stroke volume beyond heart rate. In our population, the change in cardiac output appeared to be driven by heart rate and likely represents a compensatory response to the drop in systolic blood pressure. This observation is consistent with prior reports of reduced heart rate augmentation with standing [31] and could suggest an autonomic etiology for OH, whereby the beta adrenergic response is inadequate to overcome drop in systolic blood pressure. In these patients, it appears that cardiac output may be a manifestation of autonomic dysfunction rather than a causal determinant of OH. However, we speculate that in other patients with valvular disease, beta-blockade, or heart failure, [5, 24, 25, 26, 28] absent changes in cardiac output could exacerbate fluctuations in blood pressure. Note that while not significant, our data suggested that beta blocker users may have a lower rise in blood pressure upon standing. Our findings illustrate how standing focused TTE could be a useful tool in elucidating mechanisms behind orthostatic changes in cardiac output and further etiologies of OH itself.

Our study has several limitations. First, due to the pilot nature of our study, our sample size was relatively small and we only asked a few questions of patients to minimize their burden. As a result, our ability to adjust for confounding factors was limited. Second, we only measured supine and standing BP a single time. Prior work suggests that an average of measurements in the supine and standing position could better identify clinically relevant OH [32]. Moreover, OH was assessed immediately after standing. While this has been shown to detect more cases of OH, some recommend more delayed assessments [23, 33]. Third, some safety and dizziness questions were missing for 14 participants. Nevertheless, even if these missing values were all positive for discomfort or dizziness, the overall occurrence of these symptoms was quite low. Fourth, our study was not enriched for adults with conditions or symptoms associated with OH, which should be a focus of subsequent research. Patients were not asked about an OH diagnosis during screening and changes in BP from standing were relatively small. Similarly, we did not exclude adults with autonomic forms of OH, which we suspect may have been rare. Nevertheless, future work should examine how standing TTE could differentiate between OH etiologies. Fifth, our study did not use a beat-to-beat BP monitoring device (described by others). This could provide more detailed information on the immediate BP response after standing and should be considered in future studies [34]. Finally, the relationship of antihypertensive medications with orthostatic regulation of blood pressure is of interest. However, we did not have sufficient data related to dose or timing of intake in this study. Such information would be useful for further research on the role of antihypertensive therapy and blood pressure regulation.

This study has strengths. The study population was diverse and included older adults and adults with obesity. Moreover, the protocol was successfully performed during the period immediately after standing, which can be some of the more challenging periods with respect to safety, particularly for older adults or adults with OH. Furthermore, the protocol included a concurrent assessment of BP along with the supine and standing focused TTE. Given our relatively high prevalence of OH, we were able to preliminarily examine the relationship between changes in cardiac output and OH.

These early findings show promise for assessing orthostatic changes in heart function upon standing. Little is known about cardiac function immediately after standing and current imaging modalities are traditionally performed in supine and rested positions (even post-exercise) [35]. While our protocol seemed to capture an inadequate compensatory response to systolic OH, it could also be useful for understanding other conditions that are associated with hypotensive responses (e.g., aortic stenosis and syncope). Moreover, it should help identify cardiovascular origins of OH and exculpate specific classes of antihypertensive medications from causing OH, which could inform hypertension management.

In conclusion, in this population of ambulatory patients, a standing focused TTE was efficient, safe, and related to systolic changes in blood pressure after standing. While this approach identified heart rate as the likely driver of the cardiac response in our study, future work should examine standing focused TTE in populations enriched for OH and related conditions.

## Electronic supplementary material

Below is the link to the electronic supplementary material.

Supplementary Material 1

## Data Availability

Data is not available for external use as patients did not provide consent for external data sharing.

## Abbreviations

BMI: Body mass index
BP: Blood pressure
CI: Confidence interval
CVD: Cardiovascular disease
DBP: Diastolic blood pressure
LV: Left ventricle
LVOT: Left ventricular outflow tract
OH: Orthostatic hypotension
OHTN: Orthostatic hypertension
SBP: Systolic blood pressure
