# Supplementary material for: Standing transthoracic echocardiography: a feasibility study
Source: Echo Res Pract. 2025 May 20;12:12. doi: 10.1186/s44156-025-00075-2 (PMC12090504; doi:10.1186/s44156-025-00075-2)
Supplement: Supplementary file 1 — Supplementary Material 1 [file 44156_2025_75_MOESM1_ESM.docx]

**Supplement Material**

**Supplement Table ST1.** Spearman’s correlations of changes in cardiac output and its contributing echocardiogram measures with changes in blood pressure

**Supplement Table ST2.** Echocardiogram measures associated with orthostatic hypotension or orthostatic hypertension

**Supplement Table ST3.** Echocardiogram measures associated with change in blood pressure

**Supplement Table ST4.** Association of orthostatic blood pressure changes with change in cardiac output

**Supplement Table ST5.** Association of antihypertensive medication classes with orthostatic changes in blood pressure and echocardiogram measures

| **Supplement Table ST1. Spearman's correlations of changes in echo parameters and left ventricular outflow diameter with changes in blood pressure, N=98** | | | | | | |
| --- | --- | --- | --- | --- | --- | --- |
|  | **Change in systolic blood pressure** | **Change in diastolic blood pressure** | **Change in cardiac output** | **Change in heart rate** | **Change in velocity time integral** | **Left ventricular outflow diameter** |
| **Change in systolic blood pressure** | 1.00 |  |  |  |  |  |
| **Change in diastolic blood pressure** | 0.59 | 1.00 |  |  |  |  |
| **Change in cardiac output** | -0.01 | -0.07 | 1.00 |  |  |  |
| **Change in heart rate** | -0.18 | -0.09 | 0.38 | 1.00 |  |  |
| **Change in velocity time integral** | 0.12 | -0.01 | 0.82 | -0.06 | 1.00 |  |
| **Left ventricular outflow diameter** | 0.18 | 0.02 | 0.15 | 0.10 | 0.15 | 1.00 |
| Note: Change determined based on supine minus standing measures | | | |  |  |  |

| **Supplement Table ST2. Echocardiogram measures associated with orthostatic hypotension or orthostatic hypertension, N=98** | | | |
| --- | --- | --- | --- |
| **Orthostatic hypotension** | | **OR (95% CI)** | ***P*** |
|  | Change in velocity time integral | 1.07 (0.96, 1.20) | 0.20 |
|  | Change in heart rate | 1.06 (1.00, 1.12) | 0.059 |
|  | Left ventricular outflow diameter | 1.45 (0.07, 30.23) | 0.81 |
| **Systolic orthostatic hypotension** | |  |  |
|  | Change in velocity time integral | 1.10 (0.98, 1.24) | 0.10 |
|  | Change in heart rate | 1.08 (1.02, 1.16) | 0.016 |
|  | Left ventricular outflow diameter | 1.29 (0.05, 35.33) | 0.88 |
| **Diastolic orthostatic hypotension*** | |  |  |
|  | Change in velocity time integral | 1.03 (0.86, 1.23) | 0.76 |
|  | Change in heart rate | 1.01 (0.93, 1.09) | 0.85 |
|  | Left ventricular outflow diameter | 75.00 (0.25, 22074.44) | 0.14 |
| **Orthostatic hypertension** | |  |  |
|  | Change in velocity time integral | 1.00 (0.90, 1.11) | 0.99 |
|  | Change in heart rate | 0.96 (0.91, 1.01) | 0.12 |
|  | Left ventricular outflow diameter | 3.24 (0.24, 43.88) | 0.38 |
| **Systolic orthostatic hypertension** | |  |  |
|  | Change in velocity time integral | 1.01 (0.86, 1.18) | 0.91 |
|  | Change in heart rate | 1.01 (0.90, 1.14) | 0.85 |
|  | Left ventricular outflow diameter | 56.59 (0.70, 4575.11) | 0.072 |
| **Diastolic orthostatic hypertension*** | |  |  |
|  | Change in velocity time integral | 1.02 (0.92, 1.13) | 0.71 |
|  | Change in heart rate | 0.96 (0.91, 1.02) | 0.16 |
|  | Left ventricular outflow diameter | 1.79 (0.12, 26.58) | 0.67 |
| *N=47 | |  |  |
| Adjusted for age, female | |  |  |
| *Sex predicts dependent variable perfectly, which resulted in a reduced N for regression. In a model without adjustment for sex, diastolic orthostatic hypotension the OR (95% CI) was 1.01 (0.88, 1.17) for change in velocity time integral, 1.03 (0.96, 1.11) for change in heart rate, and 0.31 (0.01, 10.35) for left ventricular outflow diameter with N=98. Note that age and sex were not associated with orthostatic hypotension or orthostatic hypertension in these models. | | | |

| **Supplement Table ST3. Echocardiogram measures associated with change in blood pressure (per 1 mm Hg), N=98** | | | |
| --- | --- | --- | --- |
|  |  | **β (95% CI) per 1 mm Hg** | ***P*** |
| **Change in systolic blood pressure** | |  |  |
|  | Change in velocity time integral | 0.18 (-0.51, 0.88) | 0.60 |
|  | Change in heart rate | -0.37 (-0.76, 0.01) | 0.057 |
|  | Left ventricular outflow diameter | 9.68 (-9.04, 28.39) | 0.31 |
| **Change in diastolic blood pressure** | |  |  |
|  | Change in velocity time integral | -0.03 (-0.52, 0.47) | 0.92 |
|  | Change in heart rate | -0.15 (-0.42, 0.13) | 0.29 |
|  | Left ventricular outflow diameter | 1.52 (-11.87, 14.90) | 0.82 |
| Adjusted for age, female | |  |  |

| **Supplement Table ST4. Association of orthostatic blood pressure changes with change in cardiac output, N=98** | | |
| --- | --- | --- |
|  | **β (95% CI)** | ***P*** |
| Orthostatic hypotension (vs. no orthostatic hypotension) | 0.60 (-0.06, 1.26) | 0.072 |
| Systolic orthostatic hypotension (vs. no systolic orthostatic hypotension) | 0.77 (0.08, 1.46) | 0.030 |
| Diastolic orthostatic hypotension (vs. no diastolic orthostatic hypotension) | 0.22 (-0.72, 1.17) | 0.64 |
| Orthostatic hypertension (vs. no orthostatic hypertension) | -0.04 (-0.69, 0.61) | 0.91 |
| Systolic orthostatic hypertension (vs. no systolic orthostatic hypertension) | -0.22 (-1.45, 1.01) | 0.73 |
| Diastolic orthostatic hypertension (vs. no diastolic orthostatic hypertension) | 0.12 (-0.54, 0.78) | 0.73 |
| Change in systolic blood pressure (supine minus standing), mm Hg | -0.00 (-0.02, 0.01) | 0.83 |
| Change in diastolic blood pressure (supine minus standing), mm Hg | -0.00 (-0.03, 0.02) | 0.73 |
| Supine systolic blood pressure per 1 mm Hg | -0.01 (-0.02, 0.01) | 0.20 |
| Supine diastolic blood pressure per 1 mm Hg | -0.02 (-0.04, 0.01) | 0.15 |
| Standing systolic blood pressure per 1 mm Hg | -0.01 (-0.02, 0.00) | 0.23 |
| Standing diastolic blood pressure per 1 mm Hg | -0.02 (-0.04, 0.00) | 0.11 |
| Adjusted for age, female |  |  |

| **Supplement Table ST5. Association of antihypertensive medication classes with orthostatic changes in blood pressure and echocardiogram measures, N=98** | | | | | |
| --- | --- | --- | --- | --- | --- |
|  |  | **Use** | **No Use** | **β (95% CI)** | ***P*** |
| **Change in systolic blood pressure, mm Hg** | |  |  |  |  |
|  | ACE/ARB (use vs no use) | 45 | 53 | 3.93 (-3.48, 11.34) | 0.30 |
|  | Beta blocker (users vs no use) | 40 | 58 | -1.78 (-9.01, 5.46) | 0.63 |
|  | Thiazide or loop diuretic (use vs no use) | 18 | 80 | -0.46 (-9.73, 8.80) | 0.92 |
|  | Calcium channel blocker (use vs no use) | 23 | 75 | 6.05 (-2.39, 14.50) | 0.16 |
| **Change in diastolic blood pressure, mm Hg** | |  |  |  |  |
|  | ACE/ARB (use vs no use) | 45 | 53 | 5.32 (0.22, 10.43) | 0.041 |
|  | Beta blocker (users vs no use) | 40 | 58 | -2.36 (-7.42, 2.69) | 0.36 |
|  | Thiazide or loop diuretic (use vs no use) | 18 | 80 | 1.60 (-4.89, 8.08) | 0.63 |
|  | Calcium channel blocker (use vs no use) | 23 | 75 | -0.17 (-6.15, 5.81) | 0.96 |
| **Change in cardiac output, mL/min** | |  |  |  |  |
|  | ACE/ARB (use vs no use) | 45 | 53 | 102.37 (-466.24, 670.98) | 0.72 |
|  | Beta blocker (users vs no use) | 40 | 58 | -52.22 (-605.38, 500.94) | 0.85 |
|  | Thiazide or loop diuretic (use vs no use) | 18 | 80 | 547.35 (-151.52, 1246.23) | 0.12 |
|  | Calcium channel blocker (use vs no use) | 23 | 75 | -562.25 (-1203.60, 79.09) | 0.085 |
| **Change in velocity time integral, units** | |  |  |  |  |
|  | ACE/ARB (use vs no use) | 45 | 53 | 0.63 (-1.56, 2.82) | 0.57 |
|  | Beta blocker (users vs no use) | 40 | 58 | 0.43 (-1.70, 2.56) | 0.69 |
|  | Thiazide or loop diuretic (use vs no use) | 18 | 80 | 2.87 (0.20, 5.53) | 0.035 |
|  | Calcium channel blocker (use vs no use) | 23 | 75 | -1.96 (-4.44, 0.52) | 0.12 |
| **Change in heart rate, beats per minute** | |  |  |  |  |
|  | ACE/ARB (use vs no use) | 45 | 53 | -2.27 (-6.20, 1.66) | 0.25 |
|  | Beta blocker (users vs no use) | 40 | 58 | -3.31 (-7.10, 0.48) | 0.086 |
|  | Thiazide or loop diuretic (use vs no use) | 18 | 80 | -1.61 (-6.52, 3.31) | 0.52 |
|  | Calcium channel blocker (use vs no use) | 23 | 75 | -1.02 (-5.55, 3.51) | 0.66 |
| **Left ventricular outflow diameter, cm** | |  |  |  |  |
|  | ACE/ARB (use vs no use) | 45 | 53 | 0.08 (-0.00, 0.16) | 0.058 |
|  | Beta blocker (users vs no use) | 40 | 58 | 0.05 (-0.03, 0.13) | 0.21 |
|  | Thiazide or loop diuretic (use vs no use) | 18 | 80 | 0.07 (-0.03, 0.17) | 0.15 |
|  | Calcium channel blocker (use vs no use) | 23 | 75 | 0.05 (-0.05, 0.14) | 0.33 |
| Abbreviations: ACE/ARB, angiotensin converting enzyme inhibitor or angiotensin II receptor blocker; CI, confidence interval | | | | | |
| Linear regression adjusted for age and female sex | | | | |  |
